# Lake bottom relief reconstruction and water volume estimation based on the subsidence rate of the post-mining area (Bytom, Southern Poland)

**DOI:** 10.1038/s41598-024-55963-0

**Published:** 2024-03-04

**Authors:** Paweł Wita, Joanna E. Szafraniec, Damian Absalon, Andrzej Woźnica

**Affiliations:** 1 University of Agriculture in Krakow, 31-120 Kraków, Poland; 2 Faculty of Natural Sciences, Institute of Earth Sciences, University of Silesia in Katowice, 41-200 Sosnowiec, Poland; 3 Faculty of Natural Sciences, Institute of Biology, Biotechnology and Environmental Protection, University of Silesia in Katowice, 40-007 Katowice, Poland

**Keywords:** Environmental impact, Geomorphology, Hydrology

## Abstract

Mining activity leads to subsidence troughs and permanent changes in water relations, like the formation of anthropogenic reservoirs. In the Upper Silesian Coal Basin (S Poland), their number is so high that the area is called an anthropogenic lake district. Any form of water retention, in the face of climate change, is valuable. However, the problem is the high variability of these lakes, making it challenging to estimate water resources. An example of this type of anthropogenic lake is the Brandka Pond in Bytom. An original method was proposed, consisting of two stages: reconstruction of the lake bottom relief based on the initial state of the area relief in 1994, i.e. at the beginning of the reservoir formation, and the land subsidence rate calculated for this area. Archival cartographic materials and DEMs from LiDAR data were used and processed in the open-source geoinformation software. Orthophoto maps and satellite scenes were also collected to determine changes in the extent of the pond from 1993 to 2019. Bathymetric data obtained in 2019 during sonar measurements on the reservoir was used to verify the calculations. The pond began to form in the early 1990s, and by 2019, it had reached an area of 178,226 m^2^, a maximum depth of 5.8 m and a capacity of 421,173 m^3^. The reconstruction method is accurate and suitable for lakes over 2 m deep, and the calculated capacity differs from the bathymetric data by 0.2%.

## Introduction

Mining activity causes one of the most significant anthropogenic transformations of the land surface (e.g.^1–9^). An example of such a region is the Upper Silesian Coal Basin (USCB) in southern Poland. Subsidence troughs are formed and developed among heaps, excavations, mounds, embankments, or numerous landforms of discontinuous deformations (sinkholes, faults, sills, fissures). Their formation proceeds about 3–6 months after the start of exploitation^10,11^. The process was influenced by the dominant method of longwall system with the roof caving giving a subsidence value of approximately 0.75–2 m for one mining horizon^12^, while mining often operates on many levels. At the beginning of the twenty-first century, the average level of exploitation was 650 m, systematically deepening by 10–15 m per year^13^. The land subsidence reached over 20 m in the long term (e.g.^13–17^), up to a maximum of 40 m^12^ and may continue to progress several years after the end of the operation.

Due to strong vertical and horizontal deformations, subsidence areas can be the epicentre of anthropogenic tremors (e.g.^18–22^). USCB is one of the most seismically active areas in the world in this respect^23^. Considering the progressing urbanization process, characteristic of USCB, it is essential from the point of view of counteracting environmental threats. Land subsidence can be a source of disaster, and cities are the areas most exposed to extreme events^24^. The limit value of the subsidence speed for the fourth category of the structural strength of buildings (category IV of mining areas) is 18 mm per day^22,25^. These speeds are sometimes exceeded in many places, especially after mining tremors. In addition, sinkholes suddenly formed; in the years 1956–1994, 53 significant landforms appeared^11^.

Geomorphologically, subsidence troughs are sedimentation basins excluded (at least temporarily) from the matter circulation. The natural sedimentation rate for the Silesian Upland area is estimated at 5–11 mm per year, using the ^137^Cs dating method for lake sediments, mainly the clay fraction, from the Brzozowice and Dąbrówka Wielka sites^26^. Along with point anthropogenic aggradation, reaching an average of 25 mm per year in 1992–2003, calculated based on the Permanent Scatterers Interferometry Synthetic Aperture Radar (PSInSAR) method for mining areas^27^, it is, however, 2–33 times lower than the amount of surface anthropogenic subsidence^14^.

Research on land subsidence and hydrological consequences uses various methods and techniques (review by^28^). Many of these methods are still based on constant monitoring of changes in land surface relief using geodetic instruments^29,30^ and satellite navigation systems^31,32^. Moreover, in many countries, including Poland, mapping of the Earth's surface is systematically carried out using airborne LIDAR data^28^ or using the photogrammetric Structure from Motion (SfM) technique^33^, which is currently experiencing a renaissance thanks to the increasing use of unmanned aerial vehicles (UAVs)^34–36^. Some of the most popular are remote sensing methods, based on obtaining information about the Earth's surface, mainly from satellite imagery, especially in the radar band (e.g.^28,37–40^). Some data are freely available in the public domain, e.g. through the browser of the Copernicus programme of the European Space Agency^41^.

Geoinformation methods facilitate research on changes in the Earth’s topography, land cover or processes (e.g.^42,43^). In this case, the Geographic Information System (GIS) may be based on the usage or the generation of digital elevation models (DEMs), which, thanks to interpolation algorithms, allow for obtaining digital terrain models (DTMs) and their derivatives. It applies to remote sensing, geodetic data, and relief reconstruction methods based on digitising contours from archival cartographic materials^44,45^. DEMs of Difference (DoDs) allow the study of land surface changes in different time intervals and with different model resolutions, depending on the accuracy of the source materials. Another type of application, important from an engineering point of view, is estimating subsidence sensitivity and risk through various indicators, classifications and predictions^46–50^.

The subject of land subsidence in Bytom has been addressed in numerous publications. Solarski et al.^44^ estimated the subsidence rate in 1883–2011 to be an average of 43 mm per year for the entire city area. For this purpose, the authors used a Prussian topographic map on a scale of 1:25,000 (Messtischblatt) with the terrain relief from 1883 and a Polish topographic map on a scale of 1:10,000, published in 1994. They rectified both maps to the modern coordinate system and then vectorised the contour lines. They created two DTMs using the natural neighbour interpolation method. The developed DTMs, through the simple algebra of raster layers, allowed for estimating terrain changes and subsidence rates in 1883–1994. The second data type was DEM, developed based on LiDAR data from 2011. Like^44^, Dulias^45^ also analysed the same archival cartographic data by morphological profiles. They allowed the author to determine the subsidence rate in the Miechowice district at 69–77 mm per year from 1881–1993, of which until 1962, it was approximately 59 mm per year, and from 1963 to 1993, it was 109 mm per year^45^.

Geodetic measurements of horizontal and vertical surface deformations, used for 60 years, indicate that in the centre of Bytom, it was, on average, 111 mm per year from 1949 to 2012 and almost 500 mm per year north of the centre in undeveloped areas (based on^15^). For 1997–1999, this rate was estimated at 100–200 mm per year based on the remote sensing method—interferometric synthetic aperture radar (InSAR)^11^. The InSAR method is commonly used to study ground deformations. It is based on radar imageries and combines signal phase shifts (differentiation of Doppler frequencies) in the echo returning from the ground surface from two slightly different positions. Research using the Differential InSAR (DInSAR) technique, i.e. comparison of two SAR images between two acquisitions, has shown that from 2009 to 2014, the subsidence rate in Miechowice ranged from 5 to 1720 mm per year^51^. Research using the DInSAR technique carried out in 2011–2012 showed that the rate of subsidence at that time reached 842 mm per year in the area of the Brandka Pond, 997 mm per year in Miechowice and 1659 mm per year in the Bytom-Karb district^12^. The quoted values show that the process of land subsidence is not uniform but strictly depends on the location of measurements within the basin, the size, depth and nature of mining, geological structure and seismic activity. A single mining tremor in the Bytom region causes a land lowering of up to 20–35 mm per day in a short period^22,23^.

Consequently, the lowering of the land surface and the inability to run off rainwater and meltwater also cause changes in water relations^17^, reaching even the third degree—the formation of anthropogenic reservoirs. Within the Katowice conurbation, their number increased from 26 to 369 from 1902 to 1994^52^. Recently, a reverse trend has been observed, as many lakes of this type are filled with post-mining waste^53^.

Although the USCB area is a part of the region called the Upper Silesian Anthropogenic Lake District with 4,473 lakes^54^, the reservoirs present here do not solve the problem of the region’s hydrological deficit. Most waters are polluted^55^, and shallow reservoirs dominate, subject to large seasonal fluctuations^56,57^. Due to organic and mineral pollution, obtaining data on the lake's bathymetry, e.g. using remote sensing methods^58,59^, may be impossible. It is not easy for such lakes to determine the surface area, especially the capacity, which is crucial from the point of view of industry needs. Another essential and desirable direction in the development of ponds in subsidence basins may be their inclusion in the blue-green infrastructure of cities or the creation of protected areas of potentially increased biodiversity^57,60–63^.

An example of an anthropogenic reservoir developed in a subsidence basin is the Brandka Pond in Bytom. Lake began functioning as a water area in the early 1990s^64^. It was influenced by the shallow exploitation of zinc and lead ores in the sixteenth century (44–96 m b.g.l.,^65^), which initiated the development of discontinuous deformations in the form of land sinkholes. The most important, however, was the start of hard coal mining in 1923 by the “Bytom” mine (later KWK “Powstańców Śląskich”).

This work aims, among others, to propose a method for estimating the capacity of an anthropogenic lake to explore water resources in terms of their future management. The first step was determining the land subsidence rate in the Brandka Pond area in Bytom from 1939/1941–2019 based on available archival cartographic materials and modern LiDAR data. These values were used to reconstruct the reservoir’s bottom relief and calculate its morphometric parameters. The method of relief reconstruction was verified using bathymetric data from 2019.

## Study area

**Figure 1:**
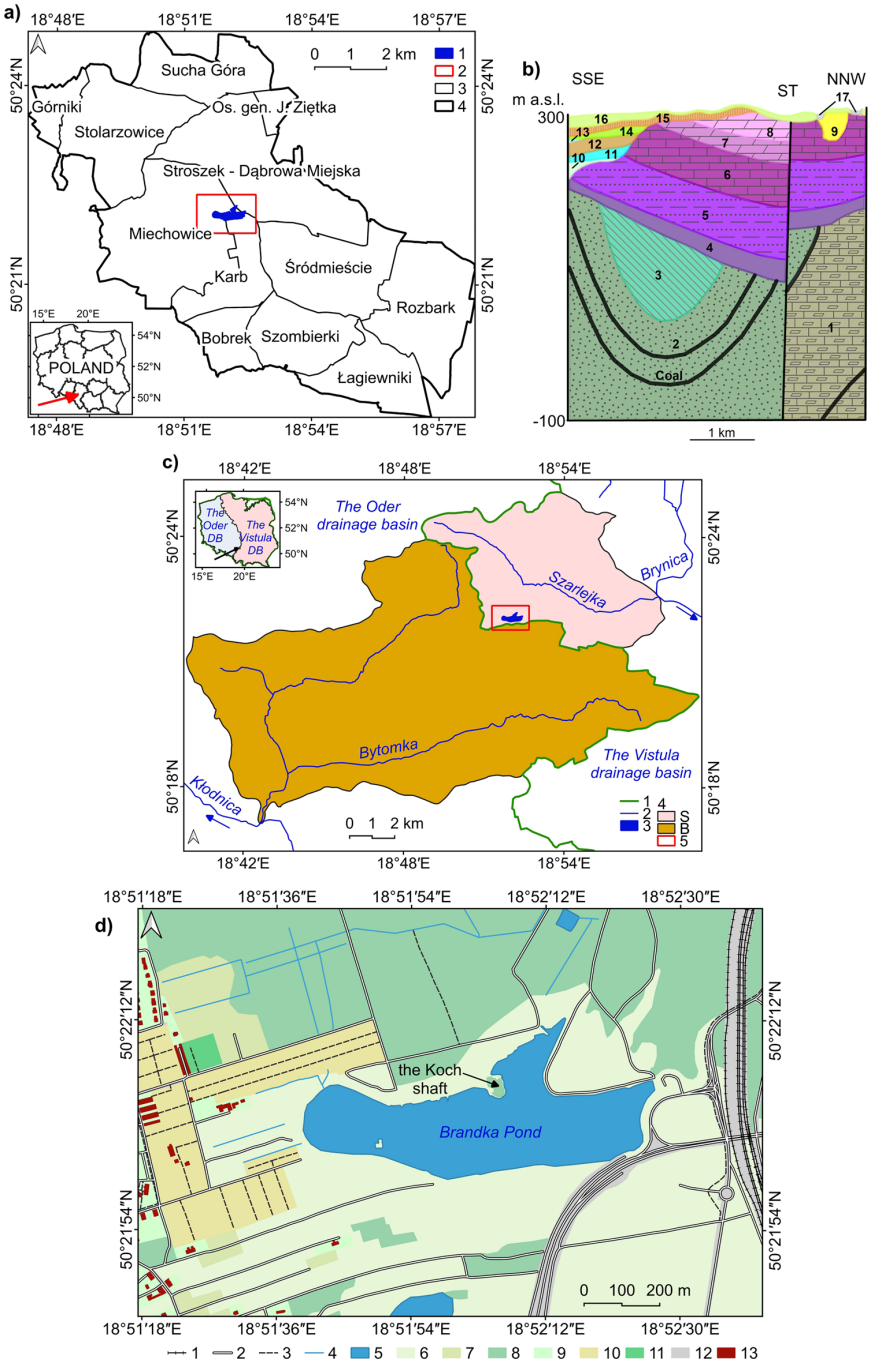
Brandka Pond and its vicinity: (**a**) the location of the Brandka Pond against the background of Bytom’s division into districts: 1—Brandka Pond, 2—study area, 3—districts, 4—Bytom; (**b**) geological cross-section of the study area cutting the Brandka Pond meridionally (based on^66^, generalised; exaggeration × 13): ST—subsidence trough; Upper Carboniferous series: 1—claystones, siltstones, sandstones and hard coal, 2—sandstones, conglomerates, claystones, mudstones, shales and hard coal, 3—mudstones, claystones, sandstones and hard coal, 4—Lower Triassic sands, sandstones, clays, claystones and siltstones, Middle Triassic series: 5—dolomites, marls and limestones, 6—limestones, 7—ore-bearing dolomites, 8—dolomites and limestones, 9—Miocene clays, loams, silts and sands, Pleistocene series: 10—river-glacial sands and gravels of the San glaciation (Mindel), 11—glacilacustrine clays and silts of the San glaciation, 12—boulder clays of the San glaciation, 13—glacilacustrine clays and silts of the Oder glaciation (Riss), 14—sands and gravels of the Oder glaciation, 15—boulder clays of the Oder glaciation, 16—fluvioglacial sands and gravels of the Oder glaciation, 17—deluvial-river sands, gravels and muds; (**c**) hydrography of the study area (data retrieved from^104^): 1—1st order water divide, 2—rivers, 3—Brandka Pond, 4—catchments: S—Szarlejka, B—Bytomka, 5—study area; (**d**) land cover of the area around the Brandka Pond (data retrieved from^69^): 1—railway, 2—road, 3—pathway, 4—river, 5—pond, 6—meadow, 7—bush, 8—forest, 9—park, square, 10—allotment, orchard, 11—cemetery, 12—trackway, 13—building. Image generated with QGIS 3.34.0 Prizren^72^.

The Brandka Pond is administratively located in the Silesian Voivodeship, in Bytom, on the border of the Karb, Miechowice and Stroszek districts (Fig. 1a). The study area and the reservoir are 1.75 km^2^, and the lake constitutes 10.7%.

Geologically (Fig. 1b), this area is built by a mudstone series and the Upper Silesian Upper Carboniferous sandstone series, cut by numerous faults^66^. These layers are also rich in hard coal deposits. Above the Carboniferous series are Triassic sands, sandstones, clays, claystone and mudstones, ore-bearing dolomites, limestones and marls (~ 180 m) with karst contact between Triassic and Miocene layers. The surface formations consist of Quaternary deposits (~ 20 m), mainly boulder clays and fluvioglacial sands and gravels of two glaciation episodes. Faults can also be seen in the formations below the Pleistocene layers, directly under the Brandka Pond basin.

Regarding hydrology, the northern part is located in the Vistula basin (Fig. 1c), the Szarlejka catchment, part of the Brynica and then the Przemsza catchment. In turn, the southern part of the area is located in the Oder basin, the catchment of Bytomka, belonging to the catchment of Kłodnica. The first-order water divide passes in the southern part of the research zone.

The land cover is varied (Fig. 1d). The northern part is occupied mainly by forests, and the southern part is by grassy vegetation. The buildings are located in the western part, with allotment gardens and orchards. In turn, the eastern part is dominated by the railway line and the Bytom ring road.

## Data and methods

### Data

Cartographic materials, aerial and satellite imageries, and digital elevation models (DEM) derived from the light detection and ranging technique (LiDAR) were used to determine the changes in the subsidence basin of the Brandka Pond in Bytom. The oldest acquired data are German topographic maps, called Messtischblatt (Ger. Meßtischblätter) on a scale of 1:25,000 (Supplementary Table S1). Since Germany adopted the Amsterdam Peil—AP elevation system in 1874 (changed to Normaal Amsterdam Peil—NAP in 1891;^67^), these maps were also made in the AP/NAP elevation system^68^. For later years, topographic maps in Polish coordinate reference systems made available via WMS services were used.

The second data group was DEMs from airborne laser scanning using LiDAR: M-34-50-D-c-4-2 and M-34-50-D-d-3-1 files. These data had a spatial resolution of 1 m and an average height error of 0.15 m and are accessible in the PL-KRON86-NH (2012) and PL-EVRF2007-NH (2019) vertical systems. They were downloaded from the Polish National Geoportal^69^.

Archival orthophoto maps (Supplementary Table S2) were collected to present changes in the shoreline of the Brandka Pond. In addition, to determine the range of seasonal fluctuations of the studied reservoir, orthophoto maps were compared with selected satellite imageries (Supplementary Table S3) available on the U.S. Geological Survey (USGS) client/server interface—EarthExplorer^70^.

### Data processing

**Figure 2:**
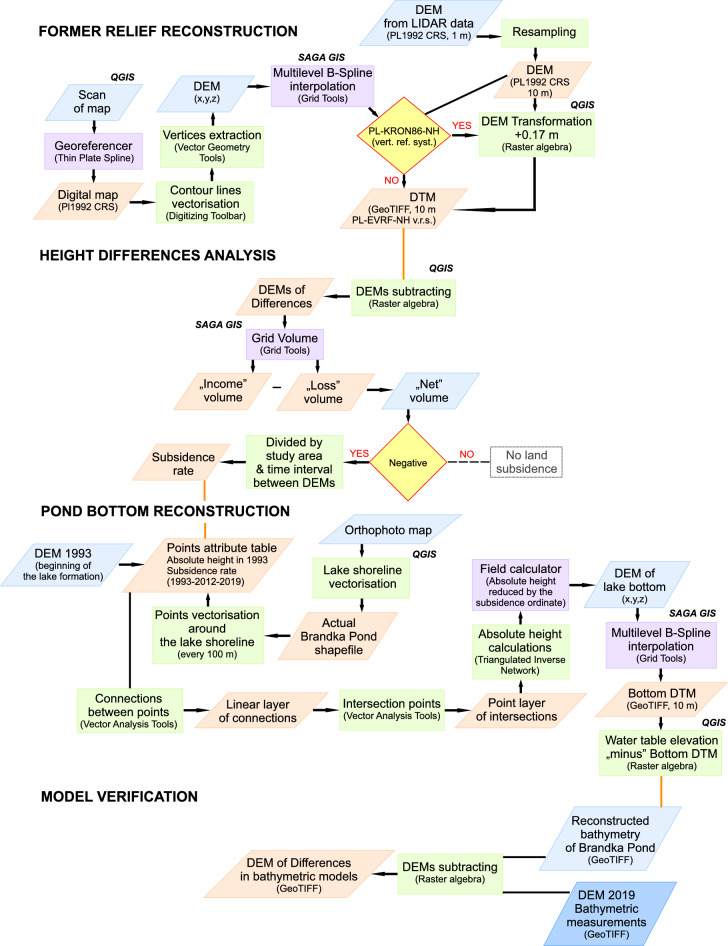
The workflow diagram presents the main stages of data processing. Image generated with CorelDRAW®X7 17.0.0.491^105^.

This part of the work consisted of several parts: reconstruction of the former terrain relief, analysis of the height differences, pond bottom reconstruction and reconstructed model verification. The main stages and data processing steps are presented as a workflow diagram (Fig. 2).

#### Reconstruction of the former relief

First, the old German maps were rectified to the Polish geodetic coordinate system PL-1992 (EPSG: 2180) in the Gauss-Krüger projection in the ETRF2000 system on the GRS80 ellipsoid. Twenty-one control points (mainly intersections and old bridges) were marked (^71^—Zenodo_Fig1.png and Zenodo_Table1.pdf), distributed relatively evenly in the Beuthen sheet, and identified on modern maps. The Georeferencer tool in open-source QGIS software under the GNU GPL licence (^72^, mainly versions 3.28 Firenze–3.34 Prizren) was used for the georeference process using the spline function method (TPS). The average transformation error for maps published in 1889, 1934 and 1943 was 1.92–4.78e^–9^. Nevertheless, it was estimated that the resulting errors might reach a value of 0.1–0.4 mm^73^, which at a scale of 1:25,000 means an error of 2.5–10 m shift. All source maps had a minimum contour line interval of 1.25 m, and contour lines were used for the manual vectorisation (Supplementary Fig. S1). Additional contours were introduced when the distances between the contour lines were large. This procedure is used primarily for relatively flat areas to increase the accuracy and correctness of the relief reconstruction already at the vectorisation stage^74^.

Among the Messtischblatts, a map from 1943 was selected. Between the maps published in 1889, 1934 and 1943, no significant topographical changes in the relief of the Brandka Pond area were found. Although it is because later maps reproduce the topography of 1881, the lack of significant changes is also evidenced indirectly by the map situation. The more important reason is, however, the annual exploitation, which started in 1923, initially in a small area, ranged from 736,000 Mg in 1929 to 1,143,800 Mg in 1938^75^, halted by the outbreak of World War II. Therefore, from 1881 to 1945, the terrain lowering was imperceptible/slowed down or less intense than after the war, with the maximum production in 1970 (2,259,490 Mg).

The number of points (14,899) in the vectorization process allowed us to generate DEM using the Multilevel b-spline interpolation method in open-source SAGA GIS software (v. 9.1.2) under the GNU GPL licence^76^ with a field resolution of 10 × 10 m. The selected interpolation method perfectly reflected the land’s topography^77^. In the case of topographic maps in the 1965 and 1942 coordinate systems (CRSs) developed in the Kronsztad elevation system, the absolute heights were transformed to the applicable PL-EVRF2007-NH (NAP) system using the correction values published by the Head Office of Geodesy and Cartography (GUGiK) (retrieved from^78^). DEMs were created for 1939/1941, 1958/1961, 1983 and 1993 (^71^—Zenodo_DEM_recon.zip).

The DEM from the LiDAR data for 2012 in the PL-KRON86-NH elevation system was transformed into the PL-EVRS2007-NH elevation system; the correction value for Bytom was + 0.17 m. DEMs from 2012 and 2019 were also resampled to a field resolution of 10 × 10 m, consistent with the values of the models reconstructed from older topographic maps.

#### Analysis of height differences

We calculated and presented the DEMs of Difference (DoDs) in the form of maps in 5 periods: 1939/1941–1958/1961 (17–22 years), 1958/1961–1983 (22–25 years), 1983–1993 (10 years), 1993–2012 (19 years), 2012–2019 (7 years). DoDs were obtained in QGIS based on simple raster algebra, similar to^44^, subtracting the older DEM from the younger one and maintaining the resolution of the input rasters, i.e. 10 m (^71^—Zenodo_DoDs.zip).

Based on DoDs, an analysis of changes in the volume of rock masses in the studied periods was carried out using the Grid Volume tool in SAGA GIS (^71^—Zenodo_Table2.pdf). The obtained differences were “net” values, allowing us to determine the size of the “loss” of rock masses—here: land subsidence. Then, the “net” values were divided by the surface area of the studied terrain. The average subsidence values of the entire area in meters were obtained. Dividing them by time showed the average rate of land subsidence per year. The changes were also presented as morphological profiles of the terrain using a Profile Tool plug-in for QGIS.

#### Pond bottom reconstruction

The DEM sourced on the 1993 topographic map was adopted to reconstruct the bottom of the Brandka Pond and as a period of its beginning. Within the contemporary range of the lake (^71^—Zenodo_Brandka_shape_2019.zip), there were very small, shallow reservoirs at that time. These conditions allowed us to assume that the bottom shape of the Brandka Pond could look similar to the relief from 1993, subject to the subsidence process.

**Figure 3:**
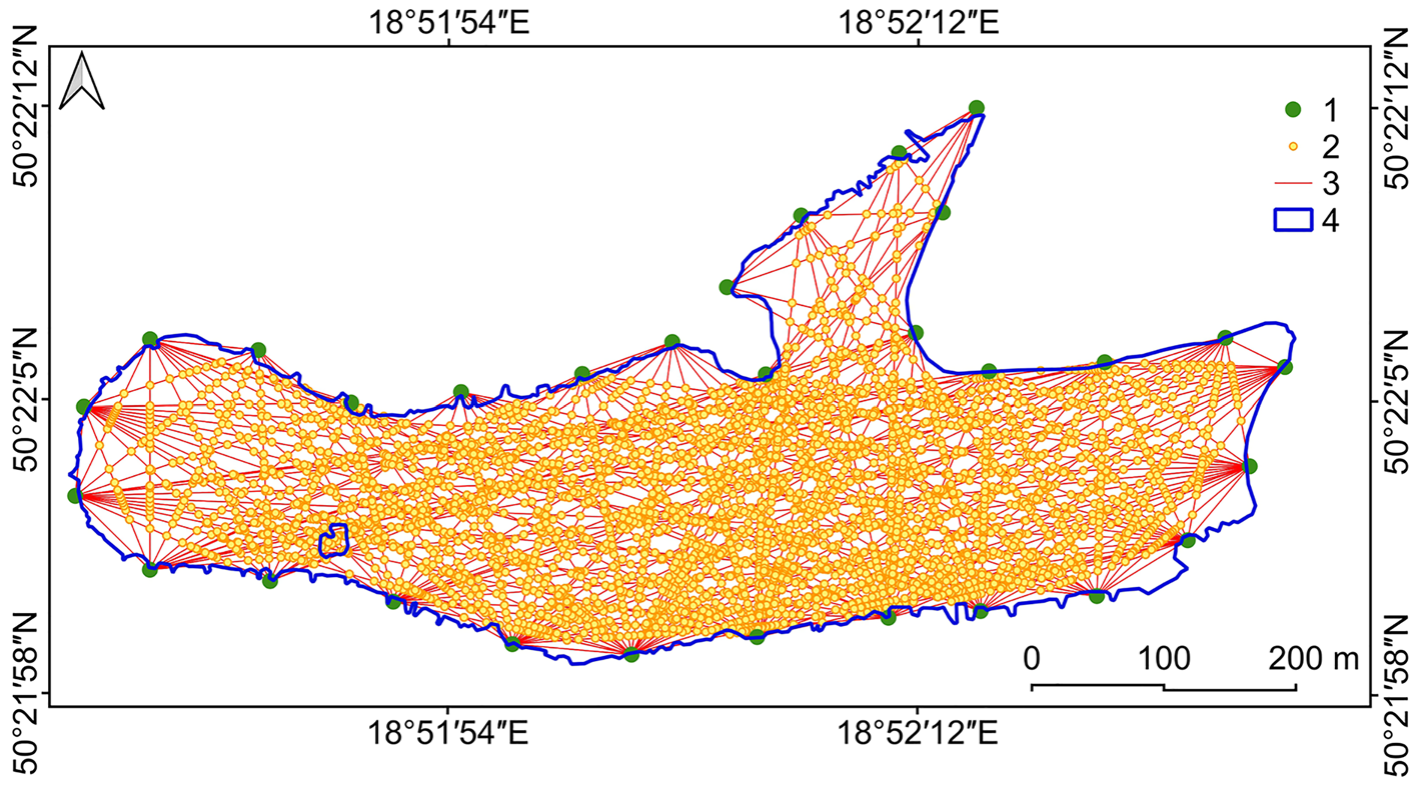
Location of the points used for the bottom relief reconstruction of the Brandka Pond: 1—points around the lake, 2—intersection points, 3—created connections, 4—Brandka Pond. Image generated with QGIS 3.34.0 Prizren^72^.

First, 30 points were determined evenly around the Brandka Pond, approximately 100 m apart and located about 3 m from the modern shoreline to represent land subsidence near a water body (Fig. 3). Then, we created connections between points as a linear layer. The next step was to determine 59,648 points at the intersections of the lines. We calculated the absolute height using the Triangulated Inverse Network method for each node on a given connection. The reference heights were data for 30 points located around the lake.

The point vector layer with fields of the absolute height in 1993 and the subsidence rate in 1993–2012–2019 allowed us to obtain the theoretical height of the terrain in 2019, i.e. “under the water surface”. It resulted from subtracting the calculated value of land subsidence for points located within the reservoir from the absolute height of the land in 1993. Then, the calculated theoretical height in 2019 under the water surface (meters above sea level) was also subtracted from the height of the water table in the reservoir in 2019. This action made it possible to obtain the theoretical depth of the Brandka Pond in 2019 (^71^—Zenodo_Brandka_reconstruction.csv).

In the next step of the reconstruction, the lake depth was interpolated in SAGA GIS with a resolution of 5 × 5 m using the Multilevel b-spline interpolation method, and the theoretical absolute height of the pond bottom in 2019 with a resolution of 10 × 10 m (^71^—Zenodo_Fig2.png).

#### Verification of the reconstructed relief of the reservoir bottom based on a bathymetric model

For this purpose, data from bathymetric measurements made by the Silesian Water Centre at the Brandka Pond on November 22, 2019, were used (^71^—Zenodo_Fig3.png and Zenodo_Table3.pdf). The tests were conducted using a sonarographic measurement set with a Lowrance HDS-5X Gen2 scanner with a single-beam HST-DFSBL transducer with 50 and 200 kHz frequencies. The scanning track was approximately 7000 m long (Supplementary Fig. S2). DEM was developed based on the obtained 10,428 point data (x, y, z) using the ReefMAster 2.0^79^ and Surfer 23^80^ programs. The coastline was determined based on the orthophoto map valid at the time of the measurement.

**Figure 4:**
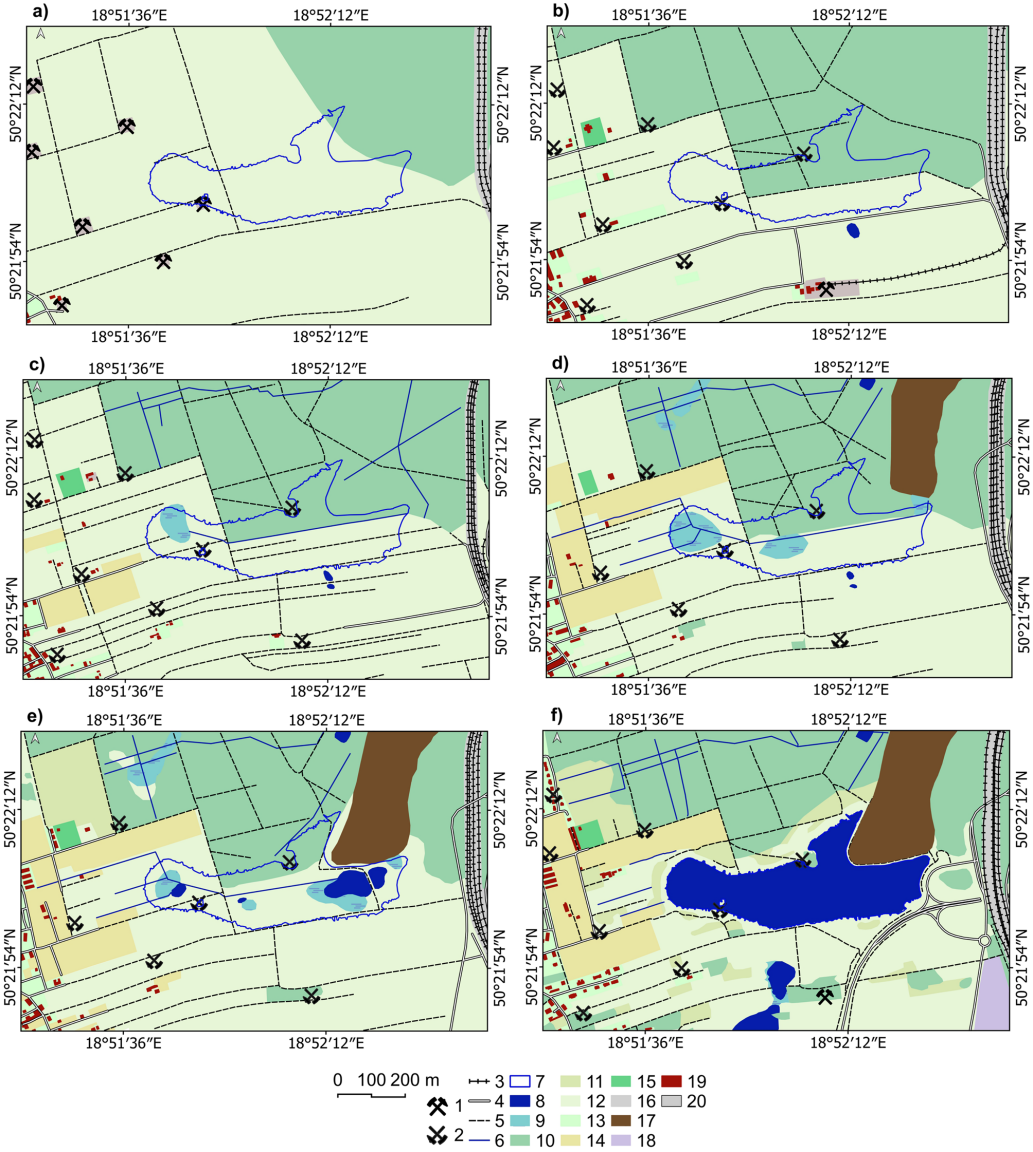
Land use of the study area in selected years from 1881 to 2019: (**a**) 1881; (**b**) 1939/1941; (**c**) 1958/1961; (**d**) 1983; (**e**) 1993; (**f**) 2019 (based on cartographic materials and the lake shoreline—based on an orthophoto map from 2019; see Supplementary Tables S1 and S2). Legend explanation: 1—mine shaft, 2—inactive mine shaft, 3—railway, 4—road, 5—pathway, 6—river, 7—Brandka range in 2019, 8—lake, 9—marsh, 10—forest, 11—bush, 12—meadow, 13—park, square, 14—allotment, 15—cemetery, 16—trackway, 17—mining heap, 18—wasteland, 19—building, 20—industrial area. Image generated with QGIS 3.34.0 Prizren^72^.

First, the DoD between the raster of the reconstructed model and the bathymetric data was calculated (^71^—Zenodo_Brandka_Depth_Difference_10m.tif and Zenodo_Fig4.png). In addition, both DEM models were used to calculate the volume of water in the pond. The absolute height of the water table 267 m a.s.l. from 2019 was adopted as the Base Level.

The verification process also used raw sonar results and depth values reconstructed at the intersection points (cf. Fig. 3). In both sets, 97 common points no more than 0.5 m apart were selected. We used the Vector selection by location tool in QGIS for this purpose.

### Changes in the surface area and land use/land cover

In addition, an analysis of changes in the surface area of Brandka Pond was carried out. A topographic map from 1993 (the beginning of the reservoir’s presence) and orthophoto maps from 1996, 2003, 2009, 2011, 2012, 2015, 2018 and 2019 were used. Whether a given orthophoto map represents the range of Brandka Pond throughout the year or whether it shows seasonal spillage, it was compared with satellite imageries from the same year (cf. Supplementary Table S3). Manual delineation of the reservoir’s shoreline was made based on these materials (^71^—Zenodo_Brandka_shapes_1993-2018.zip).

We also analysed the change in the course of the first-order watershed line between the Vistula and Oder basins, using the Convergence Index for DEM from 1993 and 2019 in SAGA GIS. In this index, maximum positive values indicate ridge skeleton lines, and minimum negative values—are watercourse/valley skeleton lines.

Changes in land use were analysed based on cartographic materials published in 1889, 1943, 1958–1961, 1983 and 1993, and orthophoto maps from 2019. Manual vectorization of particular types of land use was performed. Then, the percentage share of each land use type in the study area was calculated (^71^—Zenodo_Table4.pdf).

## Results

### Land use/land cover changes in the area of Brandka Pond in the years 1881–2019

From 1881 to 2019, there were significant changes in the land cover structure in the study area (Fig. 4;^71^—Zenodo_Table4.pdf). Since the study area is located outside the centre of the Miechowice district and land subsidence was found, no significant increase in the built-up areas was observed here. However, at the expense of grassy areas, allotments and orchards appeared in 1958/1961. In 1983, a heap of post-mining waste was built in the northeastern part. Grassland decreased from 79% in 1881 to 30% in 2019. Forested areas increased from 18% in 1881 to 33% in 2019, mainly through plant succession to grassland and the heap.

One of the significant changes in land use related to human activity is the disappearance of industrial areas due to the closure of the mining shafts. In modern times, the hills of the former mine shafts of the zinc and lead ore mines (so-called warpies) can still be seen. One of the most characteristic is the “Koch” shaft, located at the northern part of the shoreline of the Brandka Pond. When water is spilt, it creates an island in the studied reservoir.

### The land subsidence rate in the Brandka Pond area in the years 1939/1941–2019

**Figure 5:**
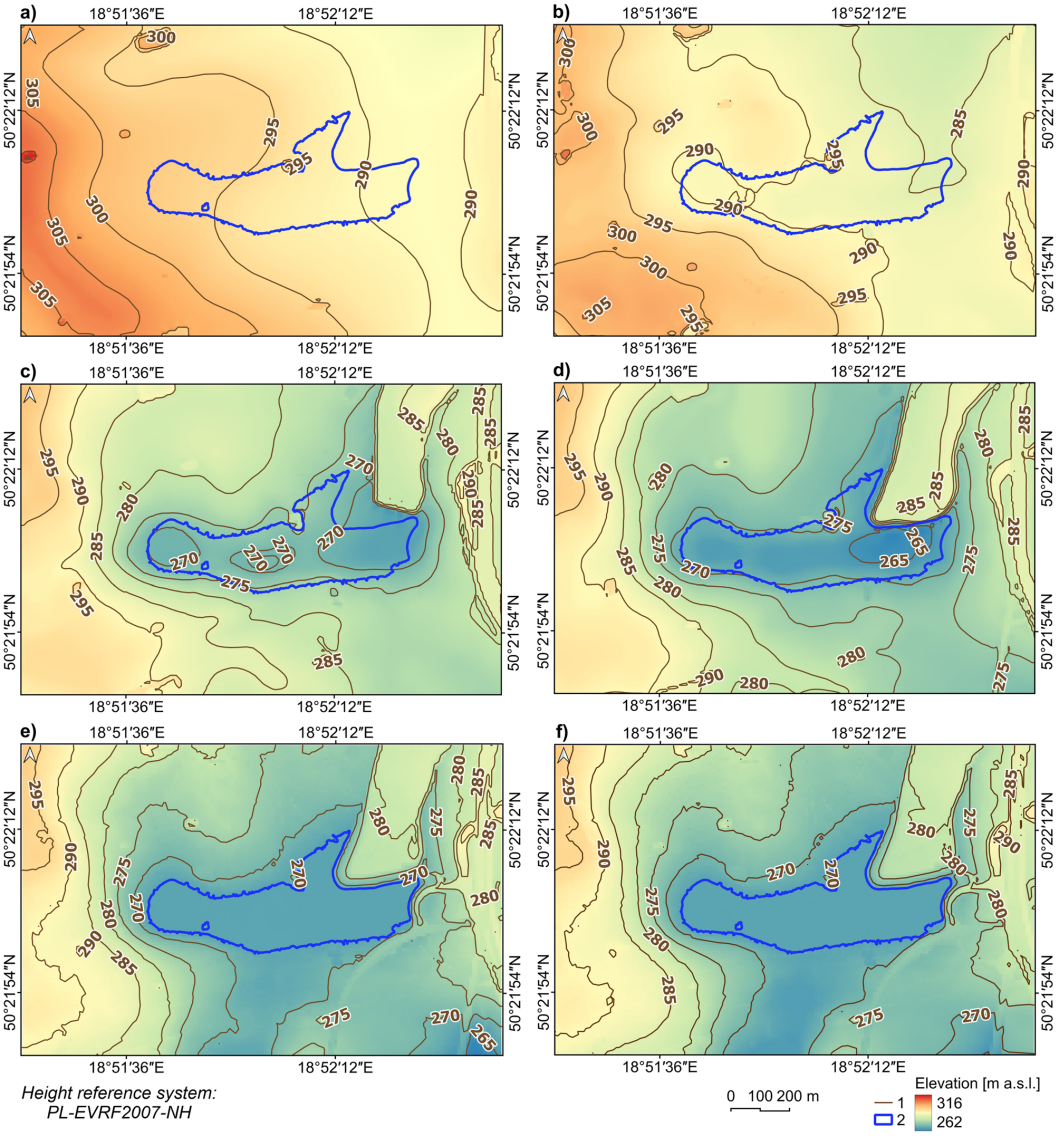
Changes in the terrain elevation near the Brandka Pond in: (**a**) 1939/1941; (**b**) 1958/1961; (**c**) 1983; (**d**) 1993; (**e**) 2012; (**f**) 2019 (based on topographic maps—see Supplementary Table S1 and DEMs from LiDAR data from 2012 and 2019^69^; the lake shoreline based on the orthophoto map from 2019). Legend explanation: 1—contour line interval 5 m, 2—Brandka Pond in 2019. Image generated with QGIS 3.34.0 Prizren^72^.

The Brandka Pond area increased from 17,000 m^2^ in 1993 (4 separate small ponds) to 178,226 m^2^ in 2019. Regarding hypsometry, in 1939/1941, the most significant percentage was occupied by areas in the altitude zone of 290–295 m a.s.l. (34.1%), followed by 295–300 (24.7%) (^71^—Zenodo_Fig5.png). The highest were areas above 315 m a.s.l., and the lowest in the altitude zone 285–290 m a.s.l. (20.6%). Over the next 78/80 years, the altitude relations were subject to significant changes, manifested, among others, in the lowering of the altitude range, constituting the largest percentage share in the research area. Nowadays, most areas are located in the altitude zone of 270–275 m a.s.l. (26.7%). Compared to 1939/1941, one can notice the disappearance of the highest altitude ranges, i.e. above 300 m a.s.l. and the emergence of new lowest altitudes (260–265 m a.s.l.). The average height of the terrain (arithmetic mean) also decreased from 295.2 (in 1939/1941) to 276.6 m a.s.l. (in 2019).

In addition to the general terrain lowering in the Brandka Pond, an uneven spatial distribution of the occurring changes was also observed (Fig. 5). In 1958/1961, the changes were mainly manifested by lowering the terrain in the northeastern part. In the following periods (1983 and 1993), significant relief transformations took place in the central part, in the place of the current reservoir. Nowadays, the evolution of the relief (2012 and 2019) is more intensive in the southern part of the study area. It is also worth noting that in 1983, a hill appeared at the northeastern borders, a post-mining waste dump that filled the earlier depression. The most significant depression of the terrain reached the value of 30.32 m in 78/80 years, which gives an average of about 379–388 mm per year. This point is located on the western shore of the Brandka Pond.

**Figure 6:**
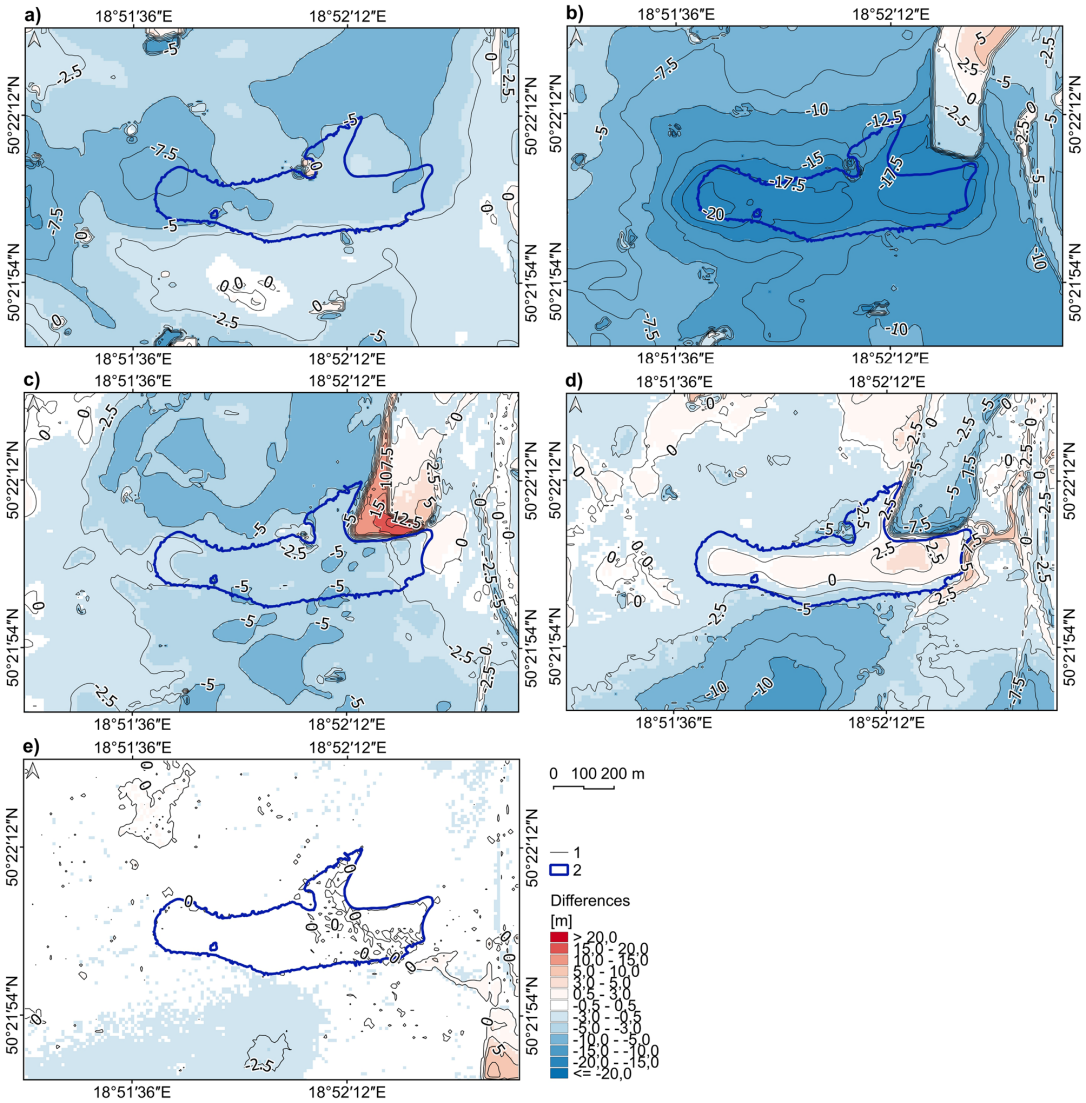
DEMs of Difference of the study area in: (**a**) 1939/1941–1958/1961; (**b**) 1958/1961–1983; (**c**) 1983–1993; (**d**) 1993–2012; (**e**) 2012–2019 (based on topographic maps—see Supplementary Table S1 and DEMs from LiDAR data from 2012 and 2019^69^; the lake shoreline based on the orthophoto map from 2019). Legend explanation: 1—contour line interval 2.5 m, 2—Brandka Pond in 2019. Image generated with QGIS 3.34.0 Prizren^72^.

The most remarkable height differences occurred between 1958/1961 and 1983 (Fig. 6). The subsidence values reached 20 m in the western part of the present reservoir. In the remaining time intervals, the land-lowering values were up to a maximum of about 10 m. Recently (2012–2019), the subsidence rate has slowed to almost zero, except for the part south of the Brandka Pond.

The obtained land subsidence values made it possible to estimate the subsidence trough’s development rate in the entire research period and in particular time intervals. Throughout the analysis period, from 1939/1941 to 2019, the study area “lost” a net volume of 32,662,028 m^3^ of rock masses (see^71^—Zenodo_Table2.pdf). This “loss” means an average lowering of the entire study area by 18.5 m, i.e. about 231–237 mm per year. The rate of lowering was an average of 170–220 mm per year in the years 1939/1941–1958/1961, 390–450 mm per year in 1958/1961–1983, 267 mm per year in the years 1983–1993, 107 mm per year in the period 1993–2012. The process almost completely stopped in 2012–2019, when the average rate of land lowering was calculated at 37 mm per year.

### The bottom relief of the Brandka Pond

**Figure 7:**
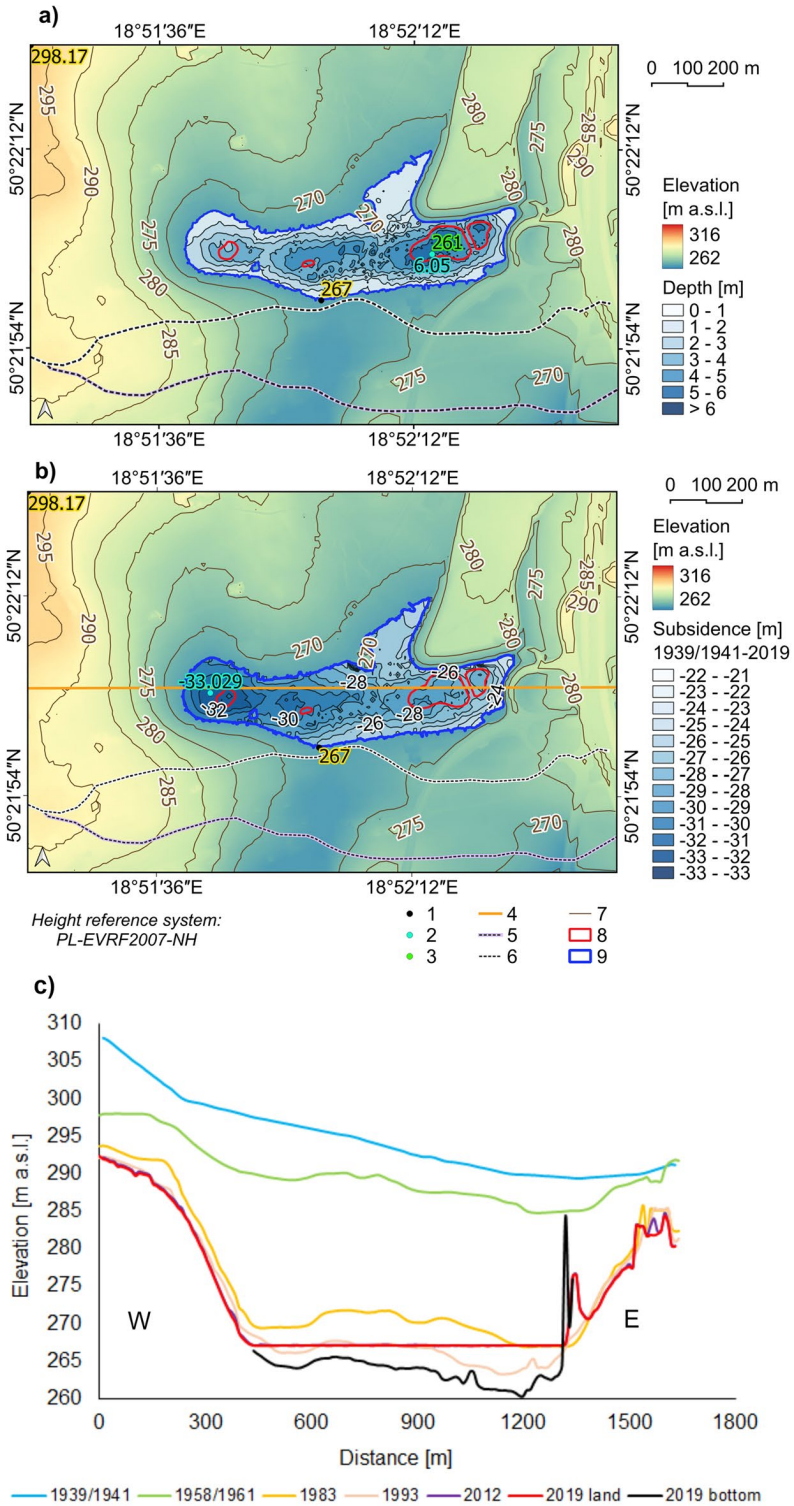
The relief of the Brandka Pond bottom and its surroundings in 2019: (**a**) the reconstructed depth of the Brandka Pond in 2019; (**b**) calculated subsidence values within the Brandka Pond from 1939/1941–2019; (**c**) latitudinal morphological profiles by the study area, taking into account the bottom of the Brandka Pond for selected years from 1939/1941–2019; vertical exaggeration × 22 (based on topographic maps—see Supplementary Table S1 and DEMs from LiDAR data from 2012 and 2019^69^, the lake shoreline based on the orthophoto map from 2019). Legend explanation (parts **a** and **b**): 1—maximum and minimum elevation, 2—deepest point/maximum of the bottom subsidence, 3—lowest point of the Brandka bottom, 4—profile line, 5—1st order watr divide line in 1958/1961, 6—1st order water divide line in 2019, 7—contour line interval 5 m, 8—Brandka Pond in 1993, 9—Brandka Pond in 2019. Image generated with QGIS 3.34.0 Prizren^72^.

The maximum depth was 6.05 m, and this point is located in the eastern part, in the outline of the initial range of Brandka Pond in 1993 (Fig. 7a). The deepest explored place is at an altitude of 261 m a.s.l., and the coastline at 267 m a.s.l. The shallowest area is a fragment of the reservoir in the form of a bay between the former Koch shaft and a mining heap. Fragments of flooded trees in this area confirm the small depth of up to 1 m.

The reconstructed depth of Brandka Pond in 2019 also allowed us to determine the subsidence value within the reservoir in the period 1939/1941–2019 (Fig. 7b). The most remarkable height differences, up to 33 m (average subsidence rate 413–423 mm per year), were observed in the western part, and the lowest, up to 22 m (average subsidence rate 275–282 mm per year), at the eastern shores of the Brandka Pond (Fig. 7c).

### The Brandka Pond extent from 1993 to 2019

**Figure 8:**
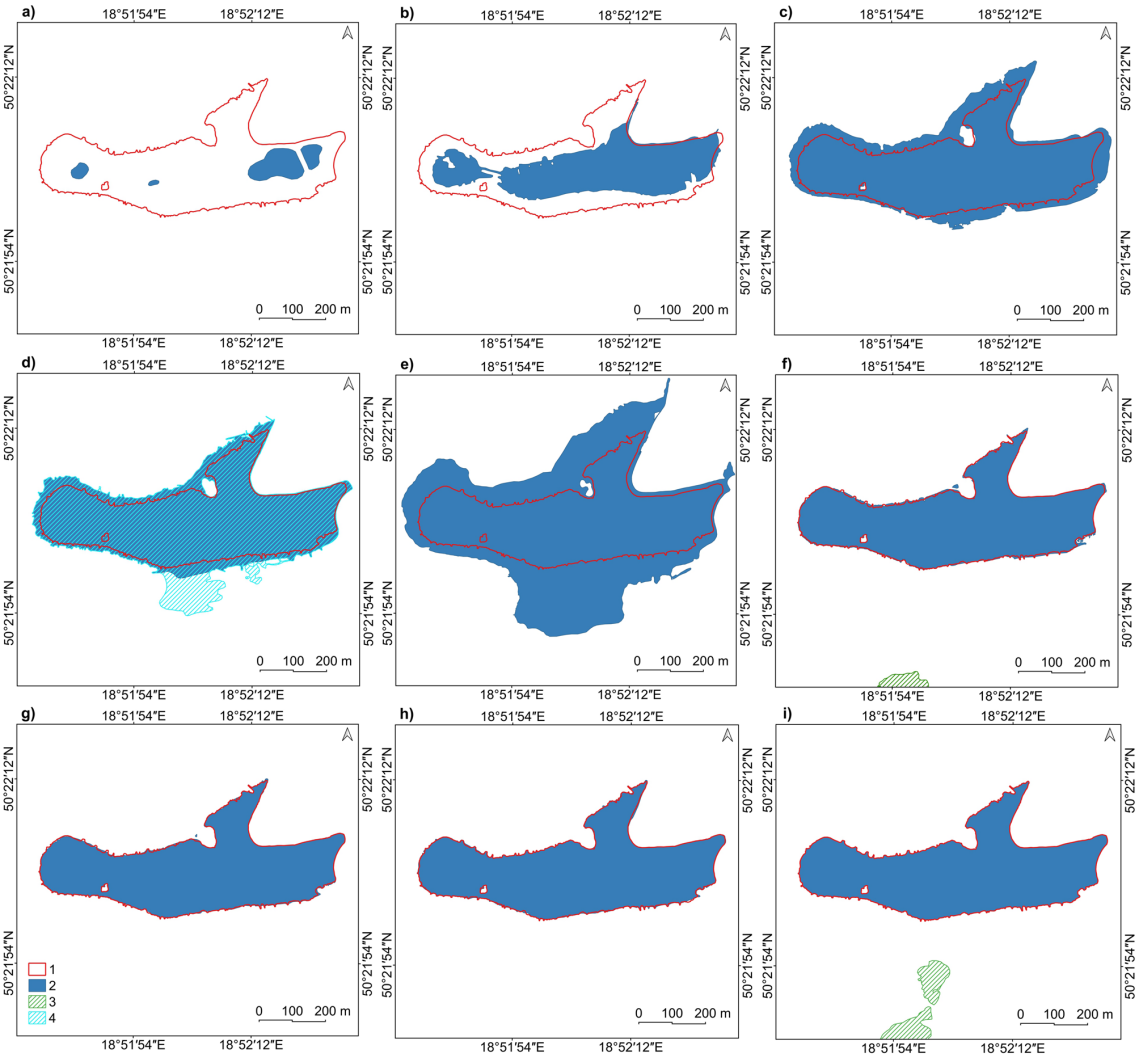
Changes in the Brandka Pond spatial range in selected years: (**a**) 1993; (**b**) 1996; (**c**) 2003; (**d**) 2009; (**e**) 2011; (**f**) 2012; (**g**) 2015; (**h**) 2018; (**i**) 2019 (based on orthophoto maps; see Supplementary Table S2). Legend explanation: 1—Brandka range in 2019, 2—Brandka Pond, 3—other lakes, 4—Brandka spillage. Image generated with QGIS 3.34.0 Prizren^72^.

Changes in the spatial extent of the Brandka Pond from its formation in 1993 to 2019 were examined (Fig. 8). At that time, the Brandka Pond increased its area by 15.6 ha. The most effective rate of surface change occurred in 2011–2012 and was associated with stabilizing the water level after spilling onto the surrounding areas in 2010. A high development rate occurred in 2009–2011 (an increase of 13.4 ha)—spillage of the reservoir water after prolonged rainfall in 2010 and in the initial phase of the reservoir development—1993–1996 (an increase of 8.2 ha). The lowest rate of changes in Brandka Pond can be observed in 2012–2019, on average ± 0.2–0.3 ha of inter-annual fluctuations, indicating a temporary shoreline stabilization.

The stabilization of the shoreline range characterizes contemporary periods. However, on the southern side of the reservoir, the adjacent floodplain in the subsidence basin—WS-47 “Bączek”—begins to develop clearly.

### Verification of the reconstructed bottom relief of the Brandka Pond—the use of a bathymetric model

**Figure 9:**
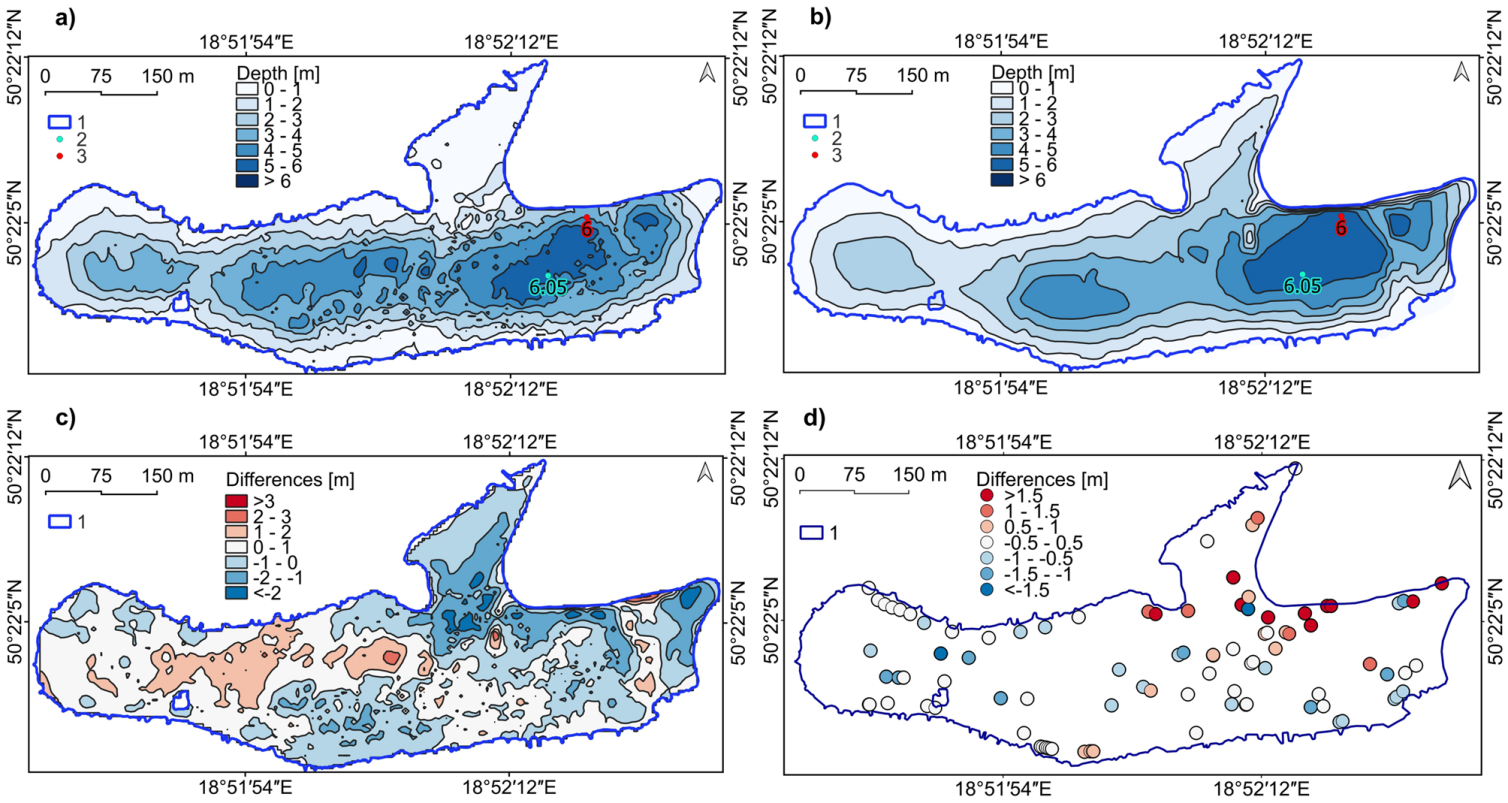
Bathymetry of the Brandka Pond in 2019: (**a**) pond depth based on the reconstruction of the bottom relief; (**b**) pond depth from bathymetric measurements in 2019; (**c**) differences between the reconstruction model and the bathymetric model; (**d**) differences between raw bathymetric measurement and the bathymetric reconstruction. Legend explanation: 1—Brandka Pond in 2019, 2—deepest point (reconstruction), 3—deepest point (bathymetric measurements). Image generated with QGIS 3.34.0 Prizren^72^.

Comparing both DEMs, obtained by the authors based on the rate of land subsidence (Fig. 9a) and bathymetric measurements (Fig. 9b), one can notice a similar course of the reconstructed isobaths to those from the actual measurements. The similarities are particularly noticeable where the bottom is trimmed in the outline of the reservoir from 1993. One of the most visible discrepancies between the former Koch shaft and the mining heap can be found in the northern part of the Brandka Pond (Fig. 9c). Also, comparing raw data from sonar surveys with the points used to reconstruct the bottom relief confirms the highest discrepancies in this area, up to approximately 2 m (Fig. 9d). Bathymetric tests by the Silesian Water Centre showed a greater depth than the reconstructed one. In addition to areas with lower values, GIS methods also showed places with greater depths, mainly in the western and central parts of Brandka Pond. The maximum depth also turned out to be slightly greater. During the bottom reconstruction test, it was determined at 6.05 m, with bathymetric measurements at 6 m.

The maximum differences between both models are − 3.84 m (shallower areas) and + 0.26 m (more deep areas), and the average is 0.23 m ± 0.88 m. The vast majority of the reservoir (70.6%) is within the depth difference range up to a maximum of ± 1 m.

The most significant negative discrepancy (shallower areas) between the former Koch shaft and the post-mining heap and in the eastern part of the Brandka Pond (from 1 to 2 m) can be observed. In turn, the most significant positive differences (deeper areas) occur in the central part of the lake (from 1 to 3 m).

The pond volume in the case of a reconstruction attempt was 422,073 m^3^ ± 370 m^3^, and according to bathymetric data, 421,173 m^3^ of water. The difference amounts to 0.2% between both models. The average depth of the pond is 2.44 m from the reconstructed model and 2.4 m from the bathymetric model.

## Discussion

The extraction of natural resources, especially hard coal, is an essential factor leading to the subsidence basins formation, and Bytom is one of the cities most affected by this process^12–16,44,51,64,81,82^. Subsidence is accompanied by mine-induced seismicity, typical of the USCB area^23,83–86^ and the formation of fault zones. It leads to an abrupt, temporary increase in the subsidence rate, exceeding the average speed by 20–30 times. The analysis showed that the general subsidence rate is not constant but varies over time, reaching maximum values during the operation period with a shift of approximately 3–6 months^11^ and then gradually slowing down. Most ground displacement occurs 1–3 years after mining stops^87^. In addition, the subsidence values are influenced by the geological structure and the mining system (especially the longwall system with caving). The more intense the rate, the more mining levels there are, and the more the analysed area is located in the centre of the subsidence basin^22^ or the fault zone^27,88^.

For the area of the Brandka Pond, mining led to the removal of output together with gangue with an average thickness of 32.5 m, giving the elevation lowering of 19 m^89^ and, according to our estimates, on average, 18.5 m. The maximum depression, considering the reservoir bottom, had the ordinate of 27.5 m in the eastern part of the pond and 33 m in its western part. Similar values (31 m) are quoted by^82^. In the analysed research area, the long-term subsidence rate reached the highest values in the period of the most intensive extraction, i.e. in the years 1960–1983, reaching an average of 430 mm per year, and it was about twice as high as the average calculated for the years 1943–2019. At that time, the average annual volume of hard coal extraction at USCB reached 200 × 10^6^ Mg in 1980 and decreased to approximately 80 × 10^6^ Mg in 2012^87^. Wagner^90^ believes the area may be stable regarding residual subsidence for approximately two years after the cessation of mining. Our studies confirmed this tendency; from 2012 to 2019, it dropped to 37 mm per year. Tajduś et al.^30^ present similar values in their summary for other countries.

One of the most important effects of the subsidence basin development is the constant change in water relations^13,17^. In extreme cases, an anthropogenic reservoir develops. The USCB area, in terms of lake content at the level of 2.74%^91^, occupies one of the top places in Poland, gaining the name of the Upper Silesian Anthropogenic Lake District. Many lakes are reservoirs in subsidence basins, such as Brandka Pond, created in the early 1990s. Its area has now stabilized at approximately 17 ha due to the excess water pumping out. The maximum depth reaches about 5.8 m and averages 2.4 m. The water volume is estimated at approximately 421,000 m^3^ (168 Olympic swimming pools), and about 429,000 m^3^ considering the pond range from 2011. However, this is a short-term situation. In 2011, the “EKOPLUS” Mining Plant was launched on the site of the liquidated coal mine “Powstańców Śląskich”^82^. This plant operates in the mining area of Bytom VII. The KWK “Bobrek-Piekary Ruch Bobrek” mine, operating in the Bytom III mining area, is also open. The resumption of hard coal mining contributes to the current land subsidence in the southern part of the research area and the expansion of the neighbouring Ws-47 lake, called Bączek. Since the Bączek Pond is developing dynamically, the Brandka Pond will probably soon capage into the Oder basin, and subsidence basins and the pond waters will merge to form a large reservoir^92^. This process is evidenced by the Brandka Pond waters pouring towards the south during the wet years (2010 and 2011). According to our estimates, the “movement” of the first-order watershed line (determined by the Convergence Index on the DEM) towards the north due to progressing subsidence south of the Brandka Pond^52,81^ is up to 3.4 m per year in the eastern part, 5.1 m per year in the south-central part of the analysis area and about 0–2.5 m per year in its western part (based on data from 1958/1961–2019; cf. Fig. 7).

In the example of Bytom, one more critical problem can be noticed. In the Karb district in August 2011, a remarkably rapid subsidence occurred, the rate of which reached 245 mm within 54 days (1656 mm per year)^12^. This event led to permanent damage to buildings, bridges and installation systems, as a result of which 140 people had to leave their buildings, and about 600 people were at risk of evacuation^93^. It occurred after a period of heavy rains, which led to numerous inundations and floods in the region, and in Bytom, it also resulted in the flooding of the Brandka Pond. This example shows how dangerous subsidence basins become in urbanized areas, where additional permanent changes in water conditions occur^17^. Combining both elements puts man in a losing position, even when calculations indicate that the building’s construction should withstand such intense subsidence.

Comparing the results of the pond bottom relief reconstruction with the data obtained using bathymetric measurements shows some differences in the depth of the reservoir, which may be partly due to errors and limitations of the adopted method. The degree of cartographic generalisation has evolved over the years. For this reason, the level of generalisation present on historical topographic maps, even those made on a similar scale, shows different characteristics compared to contemporary topographic maps^94^. Previous research indicates that the error in locating a given point on Prussian maps was 8–10 m, and the average height deviation was 0.5 m^95^. The errors were mainly due to the reproduction and printing of maps. For old maps at a scale of 1:25,000, with a graphic accuracy of up to 0.5 mm (the smallest distance between contour lines visible to the human eye), this means a 12.5 m terrain distance and a 1.25 m height difference (contour line interval). In our study, an uninhabited area with good contour lines was vectorised. Hence, a vertical error of 1.25 m is the maximum. Having rectified maps with a 300–400 dpi resolution, the contour line's width was approximately 5–9 rasters with sides of approximately 1 m of terrain value. It gives approximately 0.2–0.36 m of vertical error. Considering the above values, it can be assumed that the vertical error of the reconstructed relief of the Brandka Pond bottom could be approximately 0.7–0.9 m. The reconstruction method is not recommended for small, shallow reservoirs. In turn, reconstructions based on DEMs from LIDAR data are burdened with a vertical error of 0.15 m. An important factor affecting the accuracy of old topographic maps is also the issue of the rectification process of the scan, during which calibration errors occur. Their average value (root-mean-square error) informs the accuracy of matching the analyzed map sheet^96^. Additional uncertainty is also caused by errors related to the quality of scanned map sheets. It depends on the scanner type and the condition in which the analyzed sheet of the old topographic map was preserved^97^.

The reconstruction accuracy may be improved by increasing the number of reference points around the lake shoreline, which contain information about the “beginning” terrain height and the subsidence rate. In our study, the points were 100 m apart (cf. Fig. 3), but smaller distances, e.g. 50 or 20 m, can be used. It will increase the number of connections between the points and thus increase the number of intersections used in further calculation steps. However, the main reason for choosing the distance between reference points should be the target resolution of DEMs.

Some authors chose the Messtischblatt map published in 1889 for long-term analysis (e.g.^44,45^). The subsidence rate until the 1940s was probably low, e.g.^45^ estimates that between 1881 and 1962, the rate was, on average, 59 mm per year, and it increased only after a period of intensive exploitation. If we assume our calculations that in 1939/1941–1958/1961, the subsidence rate was about 220 mm per year, this means that between 1881 and 1943 it was, on average, about 17 mm per year. There is also the issue of the height difference of up to 0.17 m between vertical spatial reference systems (Amsterdam and Kronstadt) and their changes between the nineteenth and twenty-first centuries^67^ included in our analysis.

One possible, probably more significant difference between the models is the failure to consider the sedimentation process^98^. According to^26^ research conducted in a dried now reservoir formed in a subsidence basin, a 5–11 cm thick layer of sediments was formed during the 13–14 years of the reservoir’s operation. On this basis, the average sedimentation rate was determined at the level of 3.6–8.5 mm per year. Due to the similar genesis and the nearby location of the examined reservoir, the obtained average sedimentation rate can be related to the Brandka Pond. Considering sedimentation with an average value of 5 mm per year and the development time of Pond Brandka (26 years), there should be a layer of bottom sediments with an average thickness of about 13 cm.

The obtained value can only be treated as an estimate because the development of bottom sediments depends on many factors, both on a regional and local scale^57^. According to the research on reservoirs located in the Upper Silesian-Zagłębie region, the thickness of bottom sediments ranges from 0.2 to 179.5 cm, with an average thickness of 24.3 cm^98^. One such factor is the existence of water inflows. There are no permanent watercourses in the form of rivers or streams around Brandka Pond. However, ditches draining nearby areas and roads flow into it, providing additional material as raised, suspended and dissolved^57^. Dry and wet deposition from the atmosphere^57,99^ and surface runoff^57^ will be of greater importance in providing the material that builds bottom sediments. In this case, the proximity of the studied reservoir to the mining heap is not without significance. Due to precipitation and during snowmelt, objects of this type are washed away with fine material, which then falls to the lake bottom^100^.

The influx of pollutants, including biogenic compounds, affects the eutrophication of the reservoir, which in turn leads to its shallowing and disappearance. According to research by^101^, the Brandka Pond was classified as a eutrophic reservoir and, according to other indicators, even as hypertrophic. The pollutants supply is also confirmed by the high water electrolytic conductivity^102^. An additional element that may affect the development of the bottom sediment layer is the vegetation currently growing on the Brandka Pond banks and flooding during the development of the studied reservoir (e.g. flooded trees in the northern part, near the former Koch shaft). As a result of flooding, the vegetation gradually dies. Then, it sinks to the lake’s bottom, creating a sediment layer of organic matter^57^. It is also a reason that may cause significant differences between the reconstructed data and the data read from the sonar profile. The sonographic image is difficult to interpret, and the course of the bottom, where there is a lot of loose sediment and vegetation causing noise in reflections (see Supplementary Fig. S2), is difficult to determine. An example is the northern part of Brandka Pond, where there are submerged trees, and sonar data interpretation is challenging.

## Conclusions

Our calculations have shown that the described method, based on old maps on a scale of 1:25,000, due to the possible vertical error of ± 0.7–0.9 m, will be suitable for areas where reservoirs with a depth greater than 2 m have developed. The accuracy of the reconstruction can be, to some extent, regulated by the number of reference points around the modern pond shoreline, thanks to which we can increase the density of points for reconstruction and thus also influence the resolution of the resulting DEMs.

The analysis showed that in the case of eutrophic reservoirs, the problem in data verification is the low density of the bottom in the sonar image, resulting from the accumulated sediments and vegetation. Further field research is necessary here, requiring sampling of bottom sediments. They will improve the interpretation of sounding results and, therefore, the accuracy of the reconstructed models of the pond bottom relief.

The obtained results indicate that the reconstruction of the bottom relief based on the land subsidence rate, despite the limitations mentioned above, is a simple way to estimate the capacity of the pond and, thus, water resources for the needs of industry or blue-green infrastructure. At present, the water resources of the Brandka Pond account for about 0.4% of the annual water consumption for the industry in the Silesian Voivodeship in 2020^103^. Here, the most significant problem remains the previously mentioned issue of water purity. However, with certain expenditures and reclamation procedures, such reservoirs can also be recreational areas for the city, favour the local microclimate, mitigate the impact of urban heat islands, be included in the retention infrastructure and be a valuable area of increased biodiversity. In light of the analysis and due to the activation of coal mining, monitoring the subsidence rate of the Brandka Pond and the neighbouring Bączek Pond is necessary. Everything indicates that both lakes will soon merge, and further pumping out excess water will not be profitable. The development of this area towards including it in the blue-green infrastructure for Bytom should be a priority for the city's decision-making institutions.

### Supplementary Information

Supplementary Information.

## Data Availability

The data necessary to reconstruct the relief of the bottom of the Brandka Pond and document the evolution of the relief, the extent of the pond and land development have been placed in the international open data repository Zenodo^71^: https://doi.org/10.5281/zenodo.8209011. Other details are available in the article and in the Supplementary Tables and Figures.
