# Supplementary material for: Lake bottom relief reconstruction and water volume estimation based on the subsidence rate of the post-mining area (Bytom, Southern Poland)
Source: Sci Rep. 2024 Mar 4;14:5230. doi: 10.1038/s41598-024-55963-0 (PMC10909881; doi:10.1038/s41598-024-55963-0)
Supplement: Supplementary file 1 — Supplementary Information. [file 41598_2024_55963_MOESM1_ESM.pdf]

Lake Bottom Relief Reconstruction and Water Volume Estimation Based On the Subsidence Rate of the Post-Mining Area (Bytom, Southern Poland)

Paweł Wita, Joanna E. Szafraniec,* Damian Absalon, and Andrzej Woźnica

* Corresponding author: joanna.szafraniec@us.edu.pl

Supplementary Tables and Figures

Map	Scale	Year	Source
Messtischblatt 3309=5679 Beuthen	1:25,000	Publ.: 1889 Data actuality: 1881	Mapster – The Digital Library of Wrocław University, http://igrek.amzp.pl/details.php?id=11772869
Messtischblatt 5679 Beuthen	1:25,000	Publ.: 1934 Relief: 1881 Situation: 1929	Mapster – Western Poland Map Archive, http://igrek.amzp.pl/details.php?id=5282
Messtischblatt 5679 Beuthen	1:25,000	Publ.: 1943 Relief: 1881 Situation: 1939–1941	Mapster – Western Poland Map Archive, http://igrek.amzp.pl/details.php?id=5283
Topographic Map, the „Borowa Góra" CRS	1:5000	1958–1961	Open Regional Spatial Information System, https://geoportal.orsip.pl/image/services/MapyHistoryczne/BorowaGora_5_AP/ImageServer/WMSServer
Topographic Map, the "1965" CRS	1:10,000	1983	National Geoportal – Head Office of Geodesy and Cartography (GUGiK), http://mapy.geoportal.gov.pl/wss/service/img/guest/TOPO_SERIA/MapServer/WMSServer
Topographic Map, the "1942" CRS	1:10,000	1993	National Geoportal – GUGiK, http://mapy.geoportal.gov.pl/wss/service/img/guest/TOPO_SERIA/MapServer/WMSServer

Supplementary Table S1. Topographic maps used in the studies.

File emblem	Actuality	Pixel size [m]
M-34-50-D-c-4 RGB	1996-01-01	0.65
M-34-50-D-c-4-2 B/W	2003-01-01	0.25
M-34-50-D-c-4-2 RGB	2009-01-01	0.25
Internet Service of the Bytom Spatial Information Infrastructure, https://sitplan.um.bytom.pl/?profile=11819038	2011	0.05
M-34-50-D-c-4-2 RGB	2012-05-18	0.1
M-34-50-D-c-4-2 RGB	2015-08-11	0.25
M-34-50-D-c-4-2 RGB	2018-05-05	0.25
M-34-50-D-c-4-2 RGB	2019-04-16	0.25

Supplementary Table S2. Orthophoto maps used in the studies (retrieved from the Polish National Geoportal – Head Office of Geodesy and Cartography GUGiK, https://mapy.geoportal.gov.pl/imap/Imgp_2.html).

Scene	Acquisition date	Scene	Acquisition date
LT51890251996164FUI01	1996-06-12	LT51880252011198KIS01	2011-07-17
LT51880251996205FUI01	1996-07-23	LT51880252011246KIS01	2011-09-03
LT51880251996237FUI00	1996-08-24	LE71890252012280ASN00	2012-10-06
LE71890252003127ASN00	2003-05-07	LE71880252012241ASN00	2012-08-28
LT51890252003215MTI01	2003-08-03	LC81880252015113LGN01	2015-04-23
LT51890252003263MTI01	2003-09-20	LC81880252015305LGN01	2015-11-01
LE71890252009111ASN00	2009-04-21	LC81880252018185LGN00	2018-07-04
LE71880252009232ASN00	2009-08-20	LC81890252018288LGN00	2018-10-15
LE71890252009271ASN00	2009-09-28	LC81880252019172LGN00	2019-06-21
LT51880252011086MOR00	2011-03-27	LC81880252019284LGN00	2019-10-11

Supplementary Table S3. Landsat 5,7 and 8 satellite imageries used in the studies (retrieved from EarthExplorer, U.S. Geological Survey, https://earthexplorer.usgs.gov/).

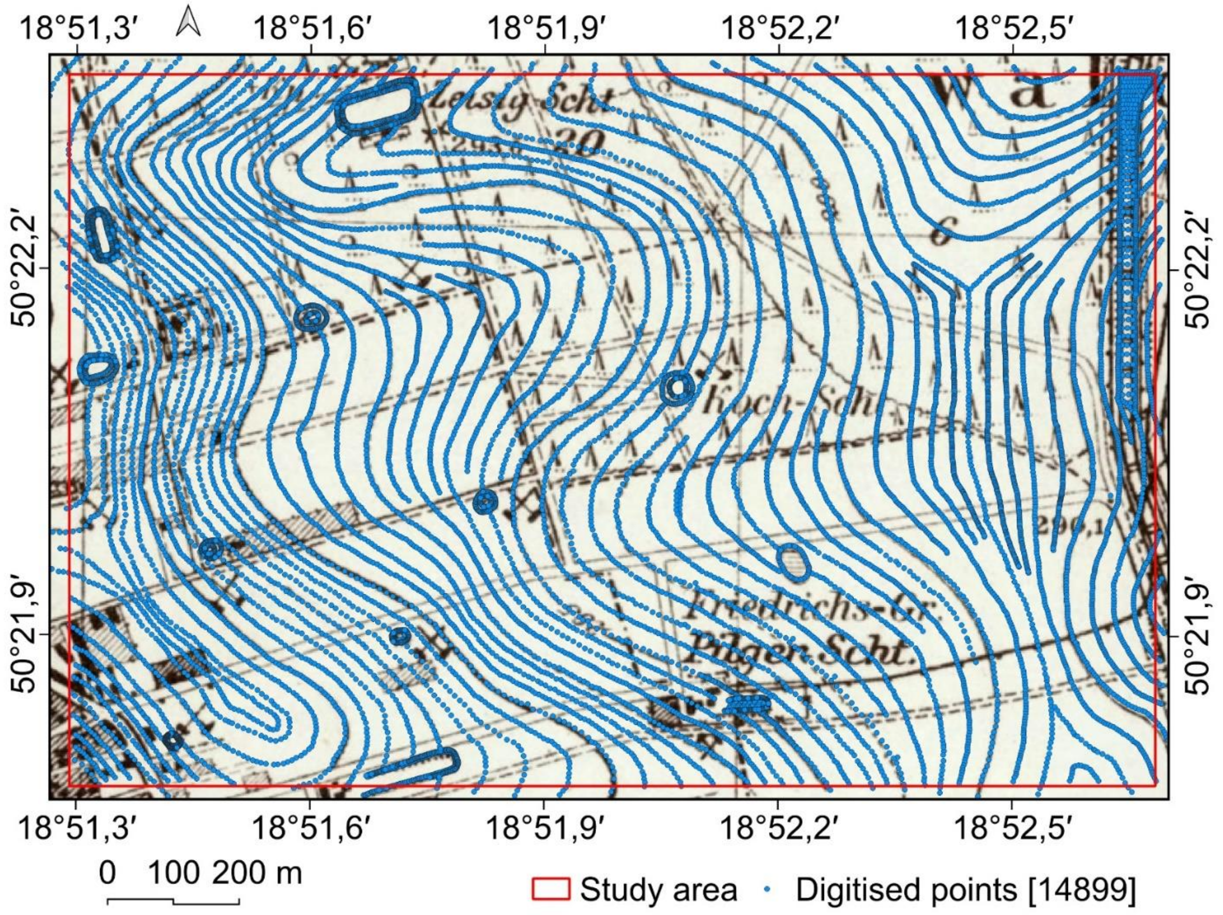

Supplementary Figure S1. An example of the digitalisation process effect along the contour lines on the Messtischblatt 5679 Beuthen from 1943 (Fragment of the background map retrieved: Mapster – Archiwum Map Zachodniej Polski => Western Poland Map Archive, http://igrek.amzp.pl/details.php?id=5283).

Statement of the Western Poland Map Archive: http://mapy.amzp.pl/faq.shtml:

In Polish: "Pozwalamy, a nawet zachęcamy do korzystania z naszych map w celach naukowych lub dydaktycznych. Można w takiej sytuacji mapę opublikować, prosimy jedynie o podanie źródła pochodzenia skanu". Translation: "We allow and even encourage the use of our maps for scientific or teaching purposes. In such a situation, you can publish the map, please only provide the source of the scan".

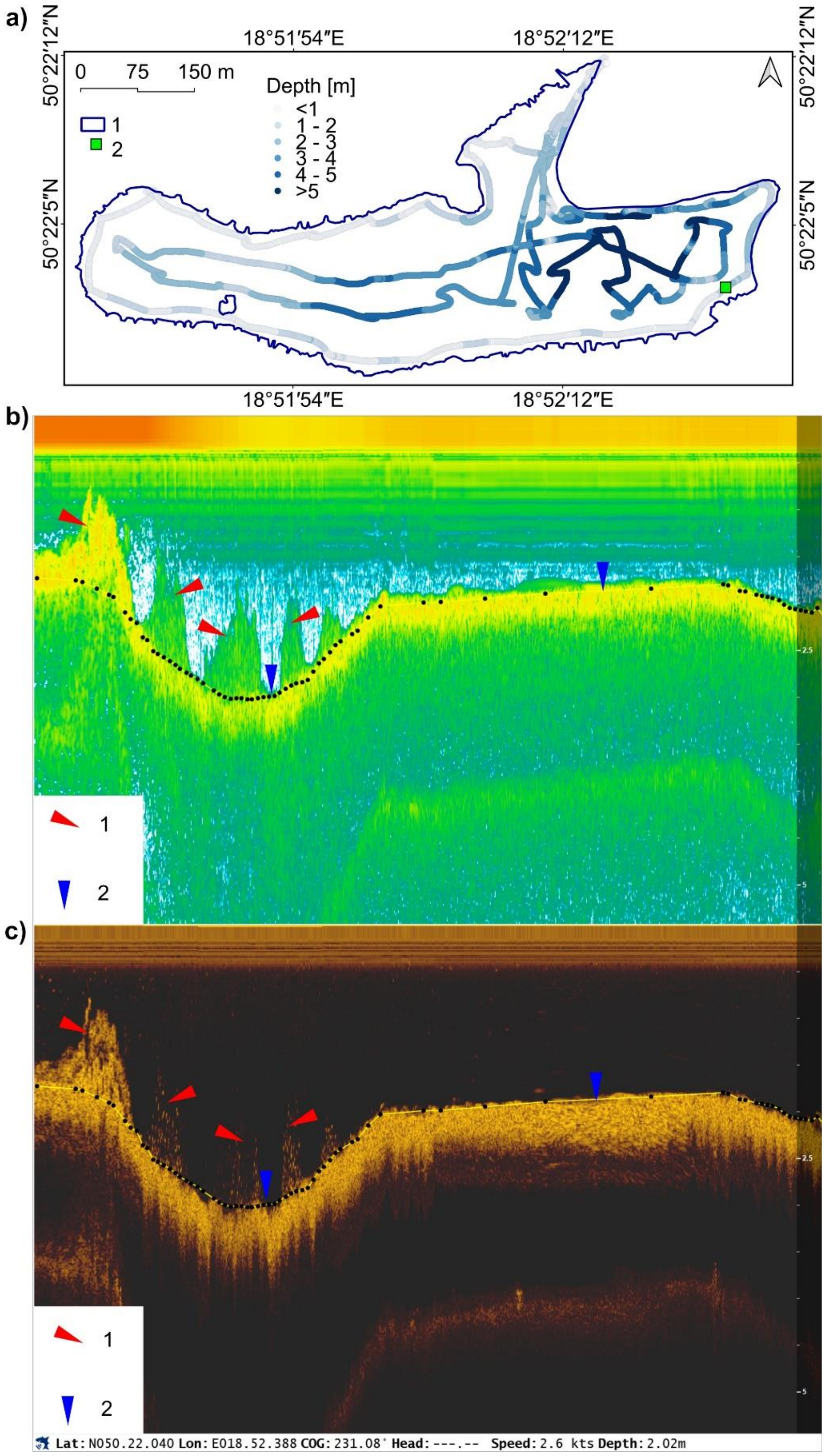

Supplementary Figure S2: Bathymetric measurements on the Brandka Pond with sonar analysis using the single beam sonar Lowrence HDS with XSONIC HDI Skimmer transducer 50/200/455/800, measurement frequency 6.6 ping s-1: **(a)** sonarografic track during depth measurements: 1 – the Brandka Pond shoreline in 2019 (based on the orthophoto map), 2 – fragment of the sonar image presented in part b and c; **(b)** scan of primary channel 63,094 pings 130-210 kHz; **(c)** Scan of downscan channel 63,017 pings, 455 kHz. Legend explanation to part b and c: 1 – submerged plants, 2 – bottom line.
